# Time taken to resume driving following hip arthroscopy

**DOI:** 10.1186/s12891-020-03662-y

**Published:** 2020-09-30

**Authors:** Suenghwan Jo, Sang Hong Lee, Se Woong Jang, Hyun Bai Choi, Ba Rom Kim, Jae Han Jeong, Soo Ah Kim

**Affiliations:** 1 Department of Orthopedic Surgery, Chosun University Hospital, Gwangju, South Korea; 2 School of Medicine, Chosun University, 365 Pilmundae-ro, Dong-gu, Gwangju, 61453 South Korea; 3 Postoperative Complication Study Group, Chosun University, Gwangju, South Korea; 4 Department of Obstetrics and Gynecology, Chosun University Hospital, Gwangju, South Korea

**Keywords:** Brake pedal depression, Brake reaction time, Driving simulator, Femoroacetabular impingement, Hip arthroscopy, Total brake time

## Abstract

**Background:**

Resuming driving is a common concern among patients undergoing hip arthroscopy. The present study aimed to assess whether patients who had undergone right hip arthroscopy presented with poorer driving performance than patients with normal hips and to analyze the time required to regain preoperative driving performance.

**Methods:**

Forty-seven patients who had undergone right hip arthroscopy and consented to our test protocol were included in this study. Using an immersive driving simulator, the patients were tested for their brake reaction time (BRT), total brake time (TBT), and brake pedal depression (BPD) preoperatively and postoperatively. The first postoperative assessments were conducted when the patients could comfortably sit on the driving seat, and the follow-up assessments were conducted for 6 consecutive weeks at weekly intervals. The patients were divided into the following two groups based on the type of surgery that they underwent: the femoroacetabular impingement (FAI) surgery group and the simple hip arthroscopy (SA) group. Twenty healthy volunteers underwent driving assessments thrice at weekly intervals and constituted the control group. The braking parameters were compared between preoperative and postoperative measurements and among the FAI surgery, SA, and control groups.

**Results:**

The preoperative braking parameters of the patients who underwent arthroscopy did not differ significantly from those of the controls (p = 0.373, 0.763, and 0.447 for the BRT, TBT, and BPD, respectively). All braking parameters returned to normal in 2 weeks in the FAI surgery group and in 1 week in the SA group.

**Conclusions:**

Our study suggests that the driving performance of patients who underwent right hip arthroscopy is comparable to that of individuals with normal hips and that the braking parameters may normalize to the preoperative state at 1 week after SA and 2 weeks after FAI surgery.

## Background

Hip arthroscopy is often indicated in young active adults, and the waiting time before resuming driving following the surgery is a common concern among the patients [1, 2]. Pain, loss of proprioception, and discomfort from the surgical intervention may result in delayed responses of the affected leg, which could potentially lead to poor driving performance [3–5]. The available literature suggests that driving performance can be recovered in 2–6 weeks following a lower leg surgical intervention, which is mostly based on the measurement of patient responses to the accelerator and the brake [1, 6]. With respect to hip arthroscopy, limited evidence is available regarding when a patient can resume driving following surgery. Similarly, there is limited information regarding whether patients with painful hips awaiting hip arthroscopic treatment show poorer driving performance.

Driving is a complex procedure, and multiple factors should be considered with respect to driving safely. The most important factor for ensuring safety while driving is an effective brake response in dangerous situations [1, 7]. As it would be unethical to test the patients’ brake responses in real-life driving scenarios, an alternative method for measuring such responses is required. Various measurement methods, ranging from the use of simple brake reaction timers or force transducers to the application of more complex realistic driving simulators, have been used in studies that provide recommendations for returning to driving [2, 7–10].

In this study, we aimed to report on the driving performance of patients who had undergone right hip arthroscopy and the time taken to recover driving performance following the surgery using an immersive realistic driving simulator. More specifically, the current study aimed to assess whether patients with painful hips awaiting hip arthroscopy have poorer driving performance than the normal population and to evaluate the time required to regain the preoperative braking performance proficiency after hip arthroscopy.

## Methods

This study was approved by our institutional review board and was registered in a prospective database (IRB#2016–08-010, KCT 0,002,643). Patients who had undergone hip arthroscopy between August 2017 and July 2019 and who fulfilled our inclusion criteria were enrolled in the study. The inclusion criteria were, as follows: 1) age between 18 and 50 years and a valid driving license, 2) history of routine commute by driving before symptom onset, 3) no influence of underlying diseases or consumption of drugs that may potential affect the driving performance, 4) history of hip arthroscopy on the right hip, and 5) provision of consent to undergo a simulator test at a weekly interval for 6 weeks. Of the 55 patients who were initially enrolled, 8 patients were excluded owing to 1) motion sickness during driving simulation (n = 2) and 2) inability to attend the entire 6-week test session (n = 6). The 47 remaining patients were classified into the following two groups based on the type of the surgery that they underwent: 1) femoroacetabular impingement (FAI) surgery group (n = 29) and 2) simple hip arthroscopy (SA) group (n = 18). Patients who underwent osteochondroplasty for a femoral head cam lesion and repair of the labrum with or without rim trimming were allocated to the FAI surgery group. Patients who underwent surgeries that did not require bone resection or labral repair and for whom the entire operation duration was < 60 min were allocated to the SA group.

We enrolled 20 healthy volunteers who were routine commuting drivers aged between 18 and 50 years and classified them as the controls (control group). The participants in the control group underwent thorough physical examination to exclude any hip problems, including range of motion, pain on movement, point tenderness, impingement test, flexion-abduction-external rotation test, and straight leg elevation test. Only the subjects who had no abnormalities during physical examination were selected.

Prior to the test, all subjects were questioned regarding their driving experience, including the number of hours spent driving each week and number of years since they acquired a driving license. For the patients who had undergone hip arthroscopy, an additional question was asked to determine if they had ceased driving or the number of hours they spent driving had decreased after the initiation of hip pain.

### Surgical procedure

All surgeries were performed by a single surgeon. The indications for surgery were as follows: 1) minimum of 3 months of conservative treatment, 2) improvement in pain with intra-articular injection, 3) detection of a pathologic lesion in radiographic images, and 4) pain severe enough to interfere with daily life. The surgeries were performed only when the diagnosis was confirmed preoperatively, which was based on the physical examination and the radiographic analysis. The patients were operated on in the lateral decubitus position, and the surgery was initiated using standard lateral and anterolateral portals. The 29 patients who underwent arthroscopy for FAI (FAI surgery group) underwent interportal capsulotomy to enhance instrument manipulation and osteochondroplasty of the femoral head with labral repair using one to three anchors. Fourteen of these patients underwent rim trimming for pincer lesions. Of the 18 patients who underwent simple arthroscopy, simple synovectomy was performed for 6 patients; labral debridement and capsular shrinkage, 5 patients; synovial chondromatosis removal, 4 patients; calcification debridement, 2 patients; and ligamentum teres debridement, and shrinkage, 1 patient. Interportal capsulotomy was performed in 15 patients from the SA group. Capsule repair was performed only when the surgeon decided that capsulotomy was excessive, which was conducted in 21 patients in the FAI surgery group and 10 patients in the SA group.

All patients were allowed to ambulate with the assistance of crutches on the first postoperative day. Range-of-motion exercises were initiated as soon as the pain was tolerable. While the patients who had undergone simple arthroscopy were not restricted from any postoperative movement, the patients who had undergone labral repair were discouraged from squatting deeply until the 6^th^ week after the procedure. For pain management, intravenous narcotics were introduced immediately following the surgery, and additional tramadol injections were prescribed according to the patients’ need. Oral pain control pills were prescribed for a minimum of 2 weeks and were re-prescribed when the patients felt it was necessary. The institution typically allows 1-week admission for patients undergoing hip arthroscopy.

### Simulator set-up and test protocol

**Fig. 1:**
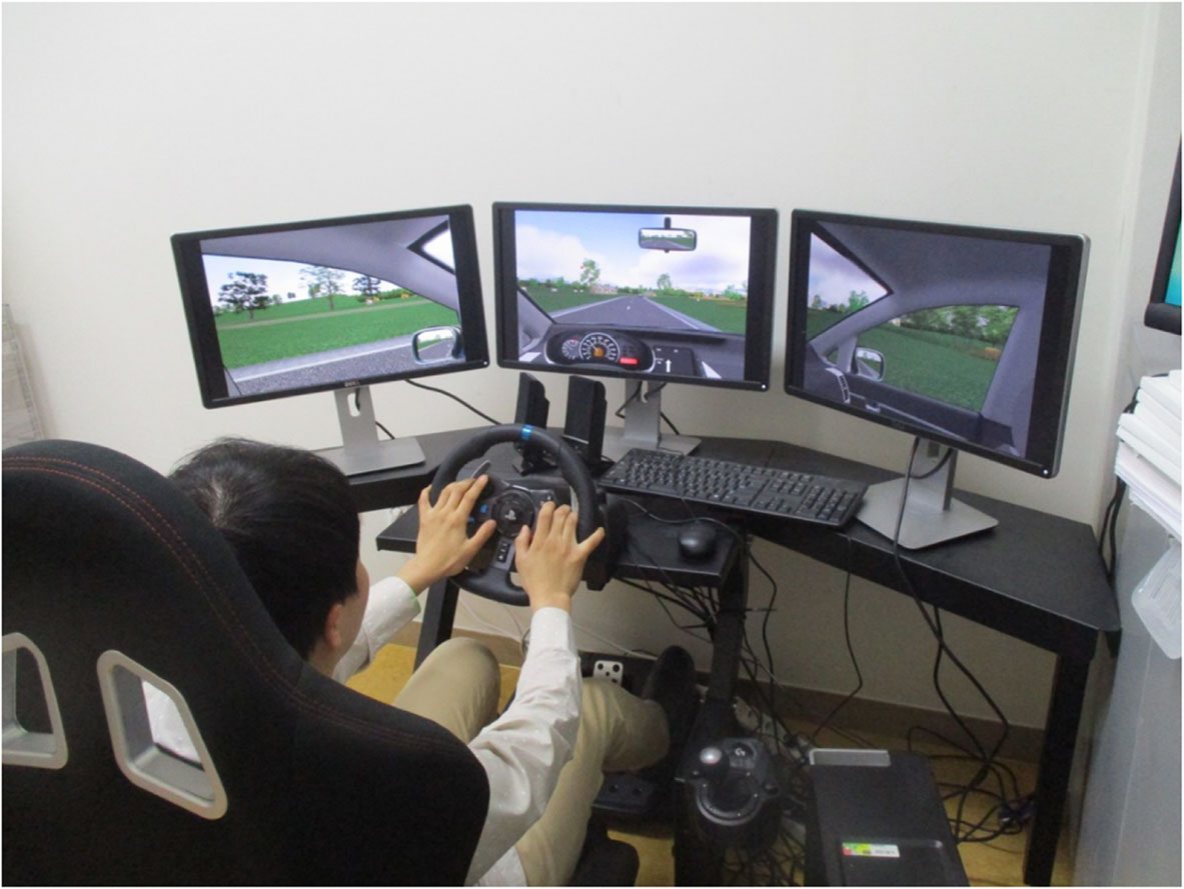
Driving simulator set up with a patient in the femoroacetabular impingement surgery group driving at 1 week postoperatively

A modern immersive driving simulator (Carnetsoft BV, Groningen, Netherlands), developed for driver training and research, was used in the current study. This driving simulator has shown high validity, excellent test–retest reliability, and significant sensitivity for testing fitness to drive [11, 12]. The simulator was composed of three monitors, a steering wheel, and a pedal unit. As the simulator was configured with an automatic transmission model, a clutch or a shifting gear was not used. Three 24-inch monitors were used for display, so that a 180-degree field of view could be provided, and an additional user interface monitor was used to control the driving scenario. The simulator provided a dashboard that included a speedometer and a tachometer. A stereo audio system, which included engine sounds and natural road traffic noises, was also provided. In addition, an adjustable driving seat was manufactured for comfortable access to the pedal unit, so that the driving simulator could imitate real driving as much as possible (Fig. 1). The test scenario, i.e., driving in a three-dimensional realistic suburban road, was developed specifically for the current study.

The test included an initial 5 min of practice driving in a suburban environment followed by a 5-min test driving session. A stopping event was described using the flashing red stop sign on the screen, which was triggered by the investigator using the separate user interface monitor. The stopping event was initiated only when the driving speed exceeded 60 km/h. Five stopping events were tested during the course of test driving.

Overall, all patients participated in eight sessions of simulated driving during the course of the study. The index driving test was performed prior to the surgery to set the baseline. Prior to the test, the patients underwent detailed instruction sessions on what to expect during the simulation. The first postoperative driving test was performed when the patients felt comfortable on sitting on the driving seat and when they felt that they are ready to attempt simulated driving. The second postoperative driving test was performed on the 7^th^ day following the surgery, and the tests were repeated at weekly intervals for 6 weeks. On the day of the simulated driving test, all patients refrained from taking opioid medication, if they had been prescribed any. The 20 healthy volunteers (control group) also underwent the same protocol, where they practiced driving for 5 min followed by 5 min of test driving. This was repeated thrice at weekly intervals to determine if acclimatization to the driving simulator may have had any potential effects on the results of the simulation.

For the outcome, the brake reaction time (BRT), total brake time (TBT), and brake pedal depression (BPD) for each of the five stopping events were measured, and the means of the five results for each parameter were used for the analysis. The BRT was defined as the time period from the first flash of the red stop sign on the screen to the patients setting their foot on the brake pedal. The TBT was defined as the time period between the stopping event and the car stopping completely. The BPD was measured as a percentage of the brake pedal pushed by the participants with respect to the pedal being fully pushed [1].

For the patient-reported outcomes, the visual analog scale (VAS) and international hip outcome tool (iHOT-12) scores were measured for all subjects prior to the test. Additionally, the VAS score was measured before each driving test being performed. All patients were recommended to not drive for 6 weeks following the surgery.

### Statistical analysis

Statistical analysis was performed using the SPSS 21 software (SPSS Inc., USA). Continuous variables were expressed as means and standard deviations (SDs). The independent t-test was performed to compare the preoperative driving performance of the patients with that of the controls, while analysis of variance (ANOVA) was used to compare the variables among the SA, FAI surgery, and control groups. The paired t-test was used to analyze the time required to regain the preoperative driving performance level after hip arthroscopy, and repeated-measures ANOVA was performed to assess whether a learning effect was noted in the control group. The sphericity of the repeated ANOVAs was tested using the Mauchly test, and Greenhouse–Geisser correction was used when the sphericity was violated. Linear regression analysis was performed to assess the correlation between the patient-reported outcomes (VAS score) and braking parameters. The significance level was set to an alpha value of 0.05 for all analyses. Sample size estimation in priori with 80% power recommended 20 subjects per group to detect a difference of 150 ms in the BRT.

## Results

The mean age of the patients who underwent hip arthroscopy was 36.2±7.9 years. No significant difference was found among the different groups in terms of age, sex, and experience in driving. At the time of surgery, three patients had completely stopped driving for a mean duration of 2.1±0.8 months prior to the surgery. Another nine patients answered that they had cut down on their driving time owing to discomfort. The demographic data and preoperative patient-reported outcomes of the patient and control groups are listed in Table 1.

**Table 1:** Demographic information of the patient and control groups

	Control group (*n* = 20)	Overall surgery cohort (*n* = 47)	FAI surgery group (*n* = 29)	SA group (*n* = 18)
Age (years)	35.2 ± 7.5	36.2 ± 7.9	37.0 ± 9.2	35.8 ± 6.4
Sex (% of women)	40	36	34	39
Driving experience (years)	7.0 ± 4.2	9.2 ± 6.3	8.8 ± 7.0	10.2 ± 5.5
Preoperative VAS score	0 ± 0.0	5.9 ± 1.0	6.2 ± 0.9	5.8 ± 1.0
Preoperative iHOT-12 score	100 ± 0.0	41.3 ± 9.4	40.8 ± 9.6	44.7 ± 8.9
Ceased or decreased duration of driving (%)	-	25	20	33

*FAI* femoroacetabular impingement; *SA* simple hip arthroscopy; *VAS* visual analog scale; *iHOT* international hip outcome tool

The driving parameters measured in the control group showed no significant difference among the three trials (p = 0.437, 0.392, and 0.543 for the BRT, TBT, and BPD, respectively), indicating that there was no learning phenomenon over the 3-week trial.

The mean BRT, TBT, and BPD of the overall surgery cohort prior to the surgery did not significantly differ from those of the control group. Moreover, no significant difference was found when these parameters were compared among the control, SA, and FAI surgery groups, indicating that the patients’ brake reaction was not influenced by the presence of hip pain in patients awaiting hip arthroscopy (Table 2).

**Table 2:** Comparison of the braking parameters between the patient and control groups

		Preoperative values in the patient groups	Control group	*P*-value^*^
BRT (ms)	Overall surgery cohort	742.2 ± 84.7	763.3 ± 75.4	0.373
	FAI surgery group	743.4 ± 78.8		0.672
	SA group	740.3 ± 94.4		
TBT (ms)	Overall surgery cohort	3341.5 ± 648.3	3250.8 ± 613.8	0.763
	FAI surgery group	3533.8 ± 616.5		0.081
	SA group	3031.7 ± 594.4		
BPD (%)	Overall surgery cohort	99.1 ± 3.6	98.4 ± 5.0	0.447
	FAI surgery group	98.6 ± 5.3		0.483
	SA group	100.0 ± 0.0		

*BRT* brake reaction time; *TBT* total brake time; *BPD* brake pedal depression; *FAI* femoroacetabular impingement; *SA* simple hip arthroscopy.

^*^The *p* value was calculated between the patient and control groups.

The patients consented to performing the first postoperative driving test at a mean duration of 3.5±1.6 days after the procedure. The patients in the SA group and FAI surgery group were able to perform the test at 3.1±1.2 days and 4.1±1.9 days postoperatively, respectively. All patients had ceased taking intravenous opioid injections by the first trial but were still taking oral pain medications. Of the 47 patients, 5 responded that they were taking pain medications at the time of the last trial, which was performed at 6 weeks postoperatively.

Compared with the preoperative state, significant prolongation of all parameters was observed in both the FAI surgery and SA groups at the first trial. At 1 week, significance was noticed only in the BRT and BPD of the FAI surgery group. No significant difference was found thereafter in both groups. Overall, the studied patients had prolonged results in all three parameters at the first trial, with the BRT and BPD remaining prolonged up to the first week (Table 3).

**Table 3:** Difference in the brake reaction parameters in comparison with the preoperative values

	FAI surgery group		SA group		Overall patient cohort	
	Mean ± SD	P-value	Mean ± SD	P-value	Mean ± SD	P-value
First BRT	-462 ± 301	**0.000**	-714 ± 402	**0.000**	-561 ± 367	**0.000**
BRT week 1	-105 ± 142	**0.007**	-59 ± 219	0.329	-87 ± 179	**0.013**
BRT week 2	-21 ± 67	0.215	+ 13 ± 73	0.521	-8 ± 70	0.660
BRT week 3	-14 ± 83	0.500	+ 5 ± 114	0.862	-7 ± 97	0.769
BRT week 4	+ 57 ± 91	0.772	+ 70 ± 111	0.516	+ 62 ± 99	0.487
BRT week 5	+ 14 ± 94	0.547	+ 16 ± 100	0.553	+ 15 ± 95	0.386
BRT week 6	+ 46 ± 94	0.806	+ 52 ± 111	0.693	+ 48 ± 100	0.639
First TBT	-540 ± 478	**0.000**	-383 ± 363	**0.002**	-480 ± 430	**0.000**
TBT week 1	+ 105 ± 332	0.209	+ 177 ± 550	0.251	+ 133 ± 437	0.090
TBT week 2	+ 150 ± 323	0.074	+ 132 ± 301	0.125	+ 143 ± 308	0.016
TBT week 3	+ 67 ± 354	0.447	+ 116 ± 252	0.108	+ 86 ± 307	0.118
TBT week 4	+ 76 ± 351	0.959	+ 94 ± 332	0.879	+ 83 ± 336	0.951
TBT week 5	+ 184 ± 481	0.134	+ 236 ± 361	0.030	+ 203 ± 424	0.011
TBT week 6	+ 237 ± 391	0.024	+ 75 ± 291	0.353	+ 175 ± 353	0.015
First BPD	+ 23.5 ± 14.9	**0.000**	+ 18.5 ± 2.3	**0.000**	+ 21.6 ± 12.5	**0.000**
BPD week 1	+ 12.3 ± 6.6	**0.000**	+ 3.6 ± 2.7	0.208	+ 8.9 ± 9.3	**0.000**
BPD week 2	+ 0.6 ± 2.4	0.332	-0.7 ± 1.6	0.671	+ 1.0 ± 4.4	1.0
BPD week 3	0.0	-	-0.7 ± 0.7	0.336	-0.3 ± 1.7	0.325
BPD week 4	0.0	-	-1.4 ± 1.4	0.336	-0.5 ± 3.5	0.325
BPD week 5	0.0	-	-1.4 ± 1.4	0.336	-0.5 ± 3.5	0.325
BPD week 6	0.0	-	-1.4 ± 1.4	0.336	-0.5 ± 3.5	0.325

*BRT* brake reaction time; *TBT* total brake time; *BPD* brake pedal depression; *FAI* femoroacetabular impingement; *SA* simple hip arthroscopy; *SD* standard deviation

The patients’ pain score (VAS score) improved from 4.9 ± 1.7 immediately after the surgery to 2.3 ± 1.2 at 6 weeks after the surgery. However, we found no correlation between the changes in the braking parameters and VAS scores throughout the test period, indicating that the postoperative pain score did not significantly influence the braking parameters (Table 4).

**Table 4:** Correlation between the VAS scores and braking parameters

	r^2^	Slope	P-value
VAS score versus BRT	0.197	0.003	0.558
VAS score versus TBT	0.178	0.003	0.164
VAS score versus BPD	0.085	-0.078	0.247

*VAS* visual analog scale; *BRT* brake reaction time; *TBT* total brake time; *BPD* brake pedal depression

## Discussion

The results of the current study indicated that driving performance, as measured by the patients’ response to braking during a driving simulation, showed no significant difference between the patients with painful hips awaiting hip arthroscopy and the asymptomatic controls. Additionally, our results indicated that the patients’ braking response recovered to the preoperative state within 2 weeks after hip arthroscopy.

The time required to return to driving following orthopedic surgery is of great concern among patients; however, only a few studies have investigated this issue [1, 6]. Endangering patients by testing their driving performance in real-life situations is highly unethical; therefore, driving simulators are used in most of the available studies on testing postoperative driving fitness. While various parameters have been used for this assessment, the ability to brake is recognized as the most important ability for safe driving [1, 6]. Currently, there is no established threshold for guaranteed safe driving; however, BRTs between 750 and 1500 ms have been suggested by various institutions [6, 13]. Using simulator data, studies have reported that patients return to average driving performance approximately 2–6 weeks after total knee arthroplasty [7, 14–16] and 4–8 weeks after total hip arthroplasty [10, 17, 18]. In contrast to hip arthroplasty, arthroscopy involves less injury to the periarticular muscle structure, resulting in less postoperative changes in strength and function. Therefore, it can be hypothesized that driving performance may be recovered earlier with arthroscopy. Studies have reported that the brake reaction function returns to the baseline levels at approximately 1–6 weeks following knee arthroscopy [8, 9, 19, 20].

To the best of our knowledge, three studies have evaluated the time required to recover fitness to drive after hip arthroscopy [2–4]. Two studies specifically assessed patients undergoing arthroscopic FAI surgery using a simple driving simulator. Vera et al. examined 19 patients who underwent FAI surgery at a 2-week interval and compared the response to braking events with that in an age- and sex-matched cohort of normal individuals [4]. The study reported that the BRT at 2 weeks postoperatively is not different from the preoperative value or that of the control. This study was largely limited by the small sample size, as it included only 11 patients who underwent right hip arthroscopy. In the study by Balazs et al., 59 patients undergoing FAI surgery were also tested at a 2-week interval [3]. However, this study did not report the number of patients undergoing arthroscopy on the right side. The study reported that patients undergoing arthroscopic FAI surgery have a prolonged preoperative BRT compared with the healthy controls and a significantly prolonged postoperative BRT, which normalized at the 4^th^ week. Conversely, Momaya et al. analyzed the patients’ braking performance using a realistic driving simulator similar to the simulator used in our study. They tested 14 patients who underwent various hip arthroscopy procedures on the right side and compared the braking parameters with those of 17 healthy volunteers [2]. The authors noticed significant improvements in the braking performance in the first 2 weeks and concluded that return to driving at 2 weeks following right hip arthroscopy is recommended. The study was limited by the different degrees of soft tissue and bone interventions, which may have influenced the braking performance, and also by its modest sample size.

Our study showed that the braking parameters normalized at 2 weeks in the FAI surgery group and 1 week in the SA group. This result is consistent with those reported by Momaya et al. and Vera et al. but conflicts with that reported by Balazs et al., who reported that a significant difference persisted at 2 weeks. The conflicting results among the studies may be attributed to the different patient rehabilitation protocols and study designs. For example, Balazs et al. instructed their patients to depress the accelerator in a midrange position until the stoplight on the screen turned red 2–10 s after the accelerator was depressed; in our study, the patients were instructed to drive at a minimum speed of 60 km/h, and the stopping events were triggered five times during the course of driving for 10 min.

Another important finding of the current study is that although FAI or hip pain may lead to impairment in the performance of daily activities, necessitating intervention, it does not lead to poor driving performance. This result is consistent with those reported by Momaya et al. and Vera et al. but again conflicts with that reported by Balazs et al. This conflict may be derived from the patients’ preoperative conditions. Our patients had mean preoperative VAS scores of 5.9 and mean iHOT scores of 41.3, which are slightly higher than those reported in the study by Balazs et al. Additionally, the mean difference in the BRT between the preoperative FAI surgery patients and the controls was 53 ms in the study by Balazs et al., which is a very small difference. The mean difference in our study was 21 ms, which we believe is a negligible difference.

While the pain score (VAS score) improved consistently postoperatively, we found that the patients’ pain score improvement did not correlate with their braking performance. We hypothesized that pain may be evoked as the patients step on the brake pedal and that this may decrease the stepping force; however, such a trend was not found. A potential reason for this may be that the pain improved significantly postoperatively, and the degree of pain experienced by the patients during the test sessions was not significant enough to influence the result.

There are several advantages of our study compared with the aforementioned previous studies. First, we classified the subjects according to the degree of soft tissue and bone procedures performed. Our results showed that the braking parameters in the SA group normalized by 1 week compared with 2 weeks in the FAI surgery group, suggesting that the degree of procedure performed on the hip joint may influence the brake response. Second, we tested the patient groups at weekly intervals. We believe that this short duration between the test sessions may provide more precise timing for the normalization of the brake response.

Simulation seems to be the only viable option for testing the patients’ fitness to drive; however, there are several limitations associated with this method. First, although we attempted to create a simulation environment as close to real driving settings as possible, driving in an actual automobile will be different, as there would be vehicular movements during actual driving. Additionally, patients will likely be more cautious when they are driving on a real road, which may have affected the results [21, 22]. Second, there is a potential learning effect, namely, the participants were introduced to simulated driving. To validate whether the potential learning effect influenced the result of the testing, the control group performed the driving test three times at 1-week intervals; our analysis showed that there was no learning effect during the 3-week trial. Third, although our study included 47 patients who underwent hip arthroscopy and was, thus, one of the largest studies in which a driving simulation test was performed, the study sample size was still relatively small. We believe a larger sample size may provide a more precise threshold for when the braking time may normalize. Similarly, our control group comprised 20 subjects which may also be relatively small. The number of control subjects was determined on the basis of the estimated sample size and expected number of recruited subjects during the study period and it is comparable with the number of participants in other published studies [2, 4, 8, 9]. However, as the braking parameters may vary in the general population, matching subjects in the control group with those in the patient group may have provided more precise results.

Although normalization of the reaction to braking may be the essential prerequisite for safe driving, driving is a complicated process, and several other factors may affect safe driving. Also, as can be seen from the large standard deviation in the postoperative brake reaction parameters, the result in the general population may vary widely and therefore the result of our study may not be applicable to all patients undergoing hip arthroscopy.

## Conclusions

Our simulation study suggests that the driving performance of patients with painful hips awaiting right hip arthroscopy is comparable to that of individuals with normal hips. Furthermore, the brake reaction parameters may return to the preoperative baseline level at 2 weeks after FAI surgery and at 1 week after SA. However, our study does not guarantee the safety of driving at this time point and applying our results in the real clinical practice may require further validation.

## Data Availability

The datasets used and/or analyzed during the current study are available from the corresponding author on reasonable request.

## Abbreviations

BRT: Brake reaction time
TBT: Total brake time
BPD: Brake pedal depression
FAI: Femoroacetabular impingement
SA: Simple hip arthroscopy
VAS: Visual analog scale
iHOT: International hip outcome tool
SD: Standard deviation
ANOVA: Analysis of variance
